# Supplementary material for: Structural basis of TRAPPIII‐mediated Rab1 activation
Source: EMBO J. 2021 May 21;40(12):e107607. doi: 10.15252/embj.2020107607 (PMC8204860; doi:10.15252/embj.2020107607)
Supplement: Supplementary file 1 — Appendix [file EMBJ-40-e107607-s004.pdf]

This Appendix contains Appendix Tables S1-S4 and their references

Appendix Table S1: TRAPP complex composition

Note: Both TRAPPII and TRAPPIII share a core set of subunits (green, middle column) and also contain unique subunits. The yeast names are shown first, with the names of the human proteins in parentheses.

TRAPPIII-specific	Shared Core Subunits	TRAPPII-specific
Trs85 (TRAPPC8)	Trs33 (TRAPPC6A, B)	Trs130 (TRAPPC10)
(TRAPPC11)	Trs31 (TRAPPC5)	Trs120 (TRAPPC9)
(TRAPPC12)	Trs23 (TRAPPC4)	Trs65
(TRAPPC13)	Trs20 (TRAPPC2)	Tca17 (TRAPPC2L)
	Bet5 (TRAPPC1)	
	Bet3 (TRAPPC3, C3L)	

Appendix Table S2: Composition of synthetic liposomes used in this study

	Golgi	Golgi no cholesterol	Anionic	No PS/PA	No PI	10% PI_4P
**PC (mol %)**	30	30	51	36	60	58
**PE**	10	10		10	10	10
**PS**	3	3	3		3	
**PA**	3	3	3		3	
**PI**	29	29	29	29		
**PI_4P**	1	1	1	1		10
**$PI_{4,5}P_2$**			0.8			
**CDP-DAG**	2	2	2	2	2	
**PO-DAG**	4	4		4	4	4
**DO-DAG**	2	2		2	2	2
**Ceramide**	5	5		5	5	5
**Cholesterol**	10		10	10	10	10
**DIR**	1	1	1	1	1	1

Note: All lipids are from Avanti Polar Lipids. PC = phosphatidylcholine, PE = phosphatidylethanolamine, PS = phosphatidylserine, PA = phosphatidic acid, PI = phosphatidylinositol, PI_4P = phosphatidylinositol 4-phosphate, $PI_{4,5}P_2$ = phosphatidylinositol 4,5-bisphosphate, CDP-DAG = cytidine diphosphate diacylglycerol, PO-DAG = palmitoyl-oleoyl diacylglycerol, DO-DAG = dioleoyl diacylglcerol

DIR = 1,1'-Dioctadecyl-3,3,3',3'-Tetramethylindotricarbocyanine Iodide

Appendix Table S3: Plasmid list

Name	Description	Vector	Source
pLT21	Trs33, Trs31, Trs23, Trs20, Bet3, Bet5	pColaDuet-1	(Thomas and Fromme, 2016)
pLT35	6xHisTEV-Mrs6	pET28	(Thomas and Fromme, 2016)
pLT40	GST-Gdi1 with cleavable tag	pGEX-6P	(Thomas and Fromme, 2016)
pLT41	6xHisTEV-Bet2, Bet4	pCDF-Duet-1	(Thomas and Fromme, 2016)
pLT50	GST-Ypt1 with cleavable tag	pGEX-6P	(Thomas and Fromme, 2016)
pLT75	mRFPmars-Ypt31 (D129N)-Fis1	pRS415	(Thomas et al., 2019)
pLT77	mRFPmars-Ypt1 (D124N)-Fis1	pRS416	(Thomas et al., 2019)
pRS415	Yeast centromeric vector with LEU2 marker	pRS415	(Sikorski and Hieter, 1989)
pSV02	mRFPmars-Ypt31 $(D129N)_{HVD}$Ypt1-Fis1	pRS415	(Thomas et al., 2019)
Ypt1-7His	GST-Ypt1-7xHis cleavable N-terminal tag	pGEX-6P	(McDonold and Fromme, 2014)
CFB2250	GFP-Atg8	pRS416	(Guan et al., 2001)
CFB2730	6xHis-Trs85[501-698]	pETDuet-1	This study
CFB2868	6xHis-Trs85, Trs33	pETDuet-1	This study
CFB2991	Trs85-mNeonGreen-3xHA	pRS415	This study
CFB3125	Trs85[R618E, R619E, R620E, K621E]-mNeonGreen-3xHA	pRS415	This study
CFB3200	Trs85[R618A, R619A, R620A, K621A]-mNeonGreen-3xHA	pRS415	This study
CFB3222	Trs85[Δ507-603]-mNeonGreen-3xHA	pRS415	This study
CFB3223	6xHis-Trs85[Δ507-603], Trs33	pETDuet-1	This study
CFB3511	mRFPmars-Ypt1 in pRS416	pRS416	This study
CFB3710	Trs85[501-698]-mNeonGreen-3xHA	pRS415	This study
CFB4191	Trs85[K378A, K381A, K382A, K386A]-mNeonGreen-3xHA	pRS415	This study
CFB4192	Trs85[K378A, F379A, F380A, K381A, K382A, F383A, K386A]-mNeonGreen-3xHA	pRS415	This study
CFB4193	Trs85[K378E, K381E, K382E, K386E]-mNeonGreen-3xHA	pRS415	This study
CFB4194	Trs85[R620A]-mNeonGreen-3xHA	pRS415	This study

CFB4195	Trs85[R620E]-mNeonGreen-3xHA	pRS415	This study
CFB4227	6xHis-Trs85[K378A, F379A, F380A, K381A, K382A, F383A, K386A], Trs33	pETDuet-1	This study
CFB4229	6xHis-Trs85[K378E, K381E, K382E, K386E], Trs33	pETDuet-1	This study
CFB4253	mRFPmars-Ypt31 (D129N)$_{HVD}$Ypt1[T181A, T182A, Q183A]-Fis1	pRS415	This study
CFB4254	mRFPmars-Ypt31 (D129N)$_{HVD}$Ypt1[K184A, K185A, E186A]-Fis1	pRS415	This study
CFB4255	mRFPmars-Ypt31 (D129N)$_{HVD}$Ypt1[D187A, K188A, N190A]-Fis1	pRS415	This study
CFB4256	mRFPmars-Ypt31 (D129N)$_{HVD}$Ypt1[V191A, N192A, L193A]-Fis1	pRS415	This study
CFB4257	mRFPmars-Ypt31 (D129N)$_{HVD}$Ypt1[K194A, Q196A, S197A]-Fis1	pRS415	This study
CFB4258	mRFPmars-Ypt31 (D129N)$_{HVD}$Ypt1[L198A, T199A, N200A, T201A]-Fis1	pRS415	This study

Appendix Table S4: Strain list

Name	Description	Source
SEY 6210	MATα *ura3-52 his3-Δ200 leu2-3,112 lys2-801 trp1-Δ901 suc2-Δ9*	(Robinson et al., 1988)
SEY 6210.1	MATa *ura3-52 his3-Δ200 leu2-3,112 lys2-801 trp1-Δ901 suc2-Δ9*	(Robinson et al., 1988)
CFY2449	SEY6210.1 Trs85-mNeonGreen::HIS3	(Thomas et al., 2018)
CFY2692	SEY6210 *trs85Δ::KanMX*	(Thomas et al., 2018)
CFY3585	SEY 6210 Trs23-6xHis-3xFLAG::TRP, *trs85Δ::KanMX*	This study
CFY3307	SEY6210.1 Bet3-GFP::HIS3, *trs85Δ::KanMX*, Trs85-mNeonGreen-3xHA:URA	This study

Appendix References

Guan J, Stromhaug PE, George MD, Habibzadegah-Tari P, Bevan A, Dunn WA, Klionsky DJ. 2001. Cvt18/Gsa12 Is Required for Cytoplasm-to-Vacuole Transport, Pexophagy, and Autophagy in Saccharomyces cerevisiae andPichia pastoris. *MBoC* **12**:3821–3838. doi:10.1091/mbc.12.12.3821

McDonold CM, Fromme JC. 2014. Four GTPases Differentially Regulate the Sec7 Arf-GEF to Direct Traffic at the trans-Golgi Network. *Dev Cell* **30**:759–767. doi:10.1016/j.devcel.2014.07.016

Robinson JS, Klionsky DJ, Banta LM, Emr SD. 1988. Protein sorting in Saccharomyces cerevisiae: isolation of mutants defective in the delivery and processing of multiple vacuolar hydrolases. *Mol Cell Biol* **8**:4936–4948.

Sikorski RS, Hieter P (1989) A system of shuttle vectors and yeast host strains designed for efficient manipulation of DNA in Saccharomyces cerevisiae. *Genetics* 122: 19 – 27.

Thomas LL, Fromme JC. 2016. GTPase cross talk regulates TRAPPII activation of Rab11 homologues during vesicle biogenesis. *J Cell Biol* **215**:499–513. doi:10.1083/jcb.201608123

Thomas LL, Joiner AMN, Fromme JC. 2018. The TRAPPIII complex activates the GTPase Ypt1 (Rab1) in the secretory pathway. *J Cell Biol* **217**:283–298. doi:10.1083/jcb.201705214

Thomas LL, van der Vegt SA, Fromme JC. 2019. A Steric Gating Mechanism Dictates the Substrate Specificity of a Rab-GEF. *Developmental Cell* **48**:100-114.e9. doi:10.1016/j.devcel.2018.11.013
