# Supplementary material for: Structural basis of TRAPPIII‐mediated Rab1 activation
Source: EMBO J. 2021 May 21;40(12):e107607. doi: 10.15252/embj.2020107607 (PMC8204860; doi:10.15252/embj.2020107607)
Supplement: Supplementary file 2 — Expanded View Figures PDF [file EMBJ-40-e107607-s003.pdf]

Expanded View Figures

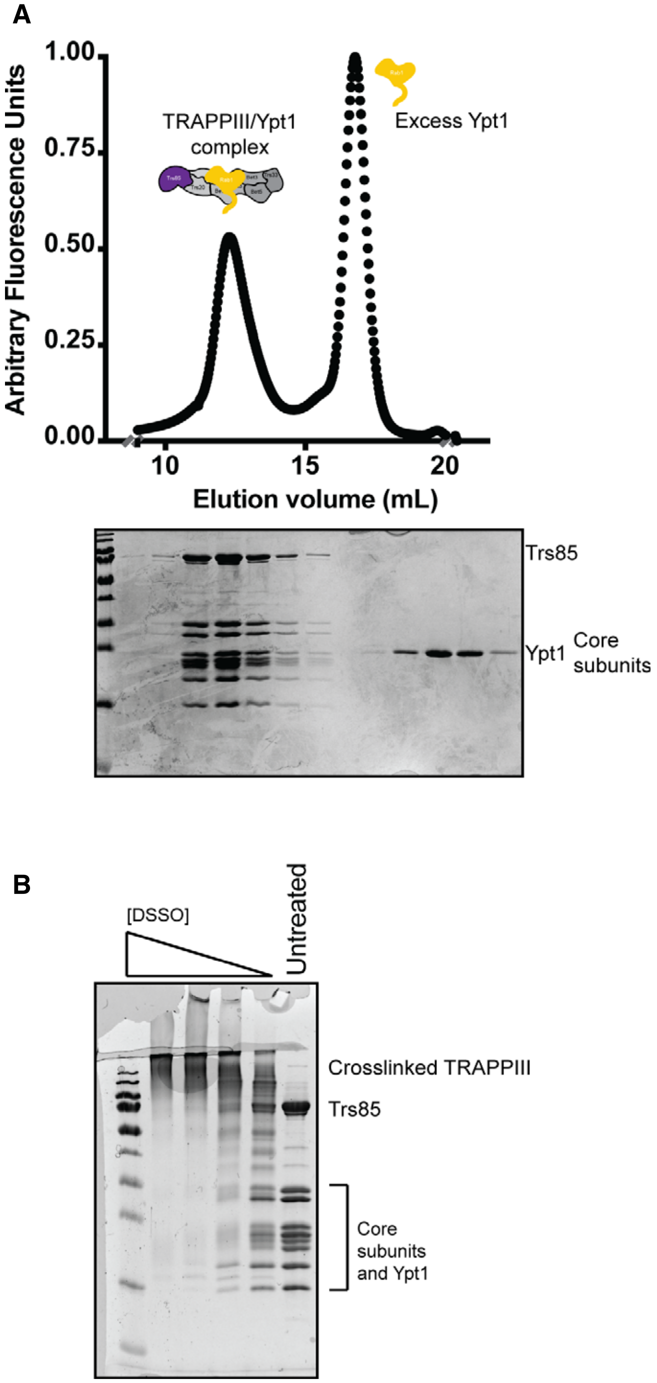

Figure EV1. Purification of the complex and cross-linking for mass spectrometry.

A Normalized UV trace from SEC to separate TRAPPIII-Rab1/Ypt1 complex from excess Rab1/Ypt1. Coomassie-stained gel corresponding to the peaks in the trace.

B Coomassie-stained gel showing the cross-linked TRAPPIII-Rab1/Ypt1 protein sample over a range of DSSO concentrations. The 625 μM sample (third lane from the right) was ultimately used for MS3 analysis and database searches.

 © 2021 The Authors

Figure EV2. Cryo-EM data.

A Example micrograph obtained from a flat dataset acquisition, 1.34 Å/pixel, −3.0 microns estimated average defocus. Scale bar, 500 Å.
B Representative 2D class averages from the flat dataset generated in RELION. The edge of each box corresponds to 482 Å. ~95% of the particles are found in the 2D classes equivalent to the Ypt1-bound class shown in Fig 5B.
C Example micrograph obtained from a 40° tilt dataset, 1.24 Å/pixel, −1.5 microns estimated average defocus. Scale bar, 500 Å.
D Representative 2D class averages from the combined 30°, 40°, and 45° tilt datasets generated in RELION. The edge of each box corresponds to 397 Å.
E FSC curves for each of the final refined particle sets used for 3D reconstructions (see also Appendix Fig S3).
F FSC curves denoting the agreement between the final model and the following maps: the unsharpened full map, the half-map used for refinement, and the half-map used for validation. The half-map/half-map FSC curve is included for comparison.
G Orientation distributions of each of the final refined particle sets used for 3D reconstructions (see also Appendix Fig S3). The heatmap represents number of particles refined to each point in orientation space. Generated using a script written by A. Leschziner available on GitHub.

© 2021 The Authors

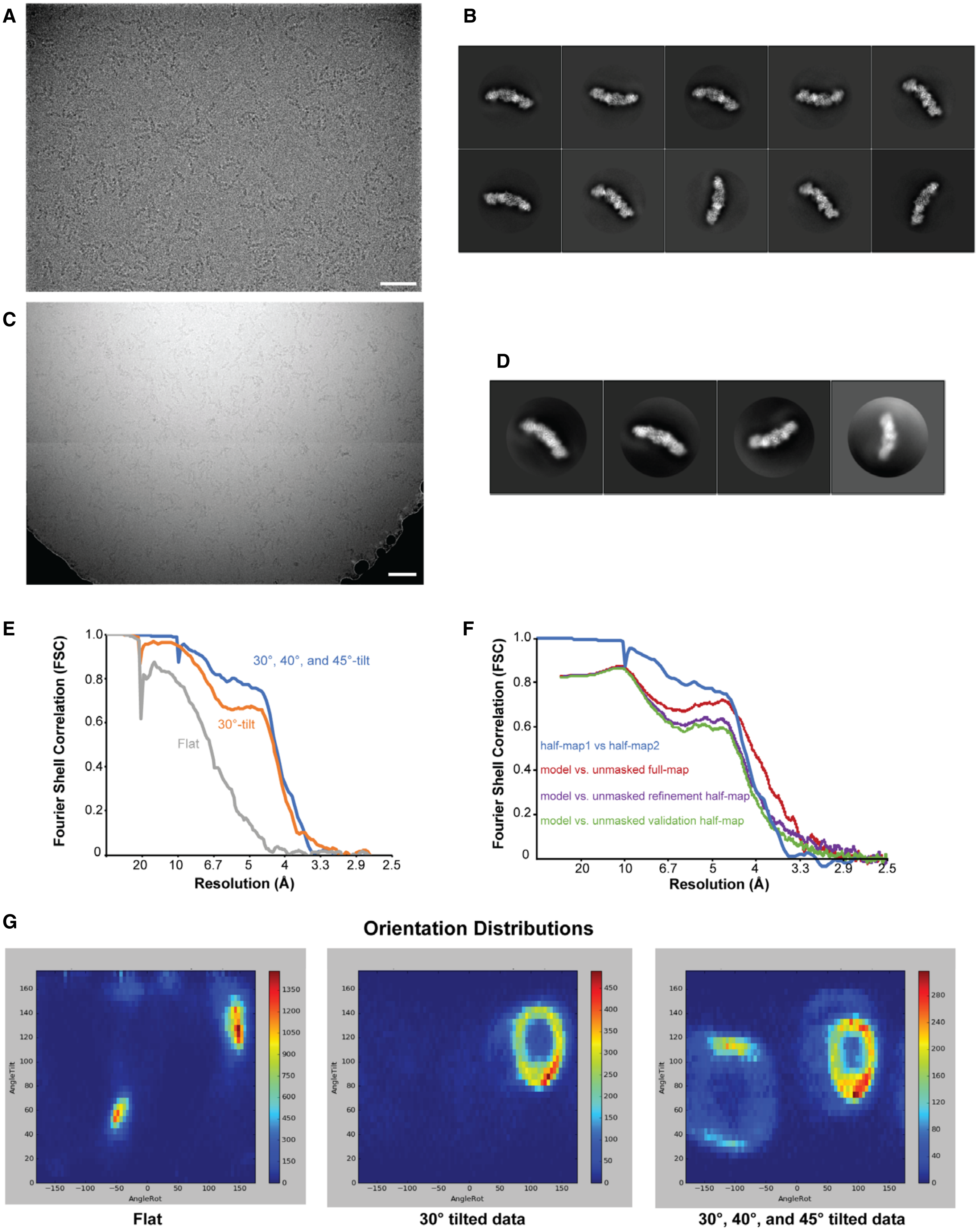

Figure EV2.

© 2021 The Authors

Figure EV3. Cryo-EM data processing flowchart.

Flow chart showing the steps taken during data processing to generate the high-resolution TRAPPIII-Rab1/Ypt1 3D reconstruction. "Sphericity" is a measure of data isotropy (lower values represent more anisotropy) and was calculated using a 3DFSC script written by D. Lyumkis available on GitHub. The insets show the EM density from each of the final 3D reconstructions, surrounding the portion of Trs85 that contacts Trs20.

© 2021 The Authors

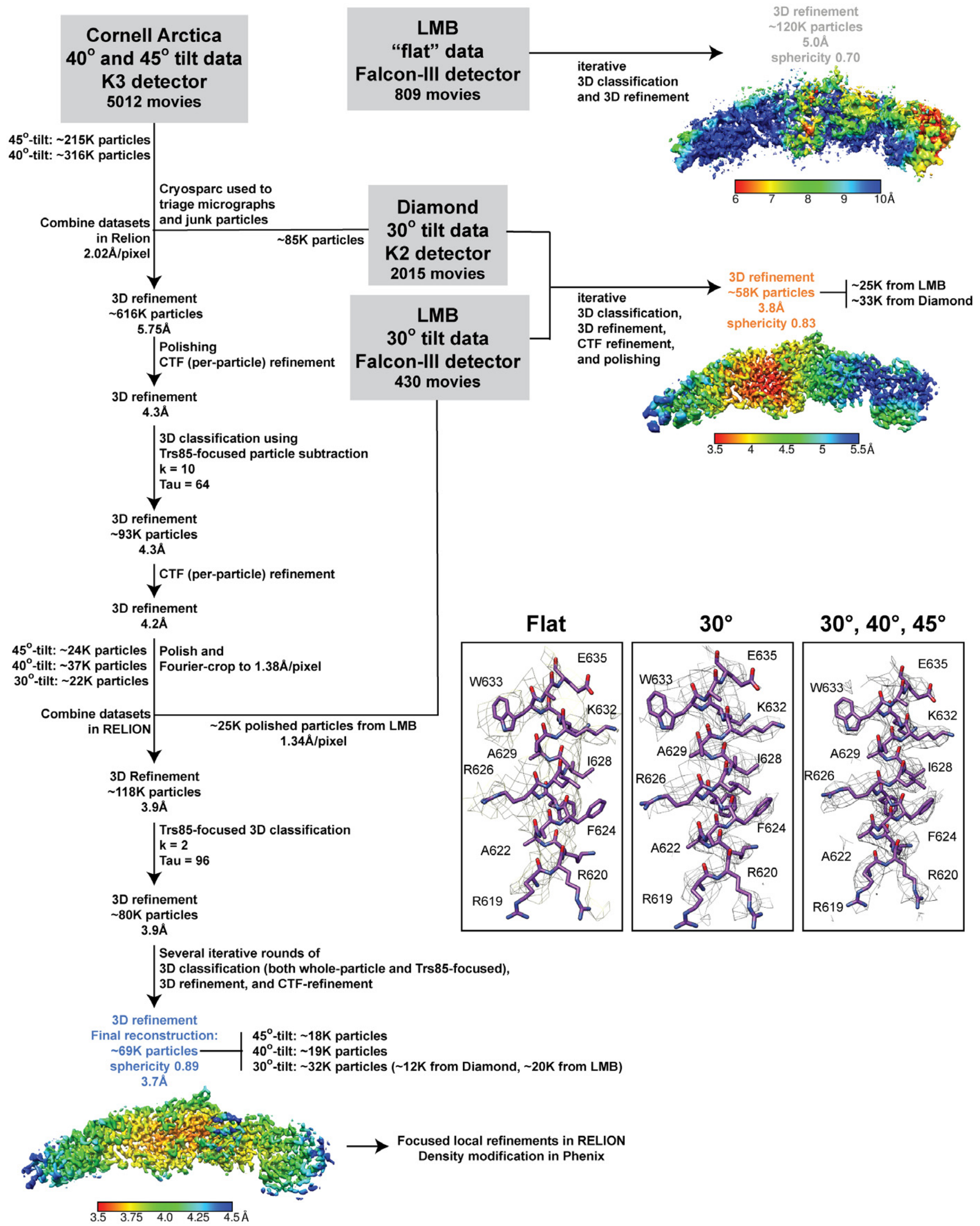

Figure EV3.

© 2021 The Authors

Figure EV4. Comparisons to published crystal structures.

A Bet3 subunit from our cryo-EM reconstruction aligned with the Bet3 subunit from pdb:3CUE.
B The second Bet3 subunit from our cryo-EM reconstruction aligned to the second Bet3 subunit from pdb:3CUE.
C Bet5 subunit from our cryo-EM reconstruction aligned with the Bet5 subunit from pdb:3CUE.
D Trs23 subunit from our cryo-EM reconstruction aligned with the Trs23 subunit from pdb:3CUE.
E Trs31 subunit from our cryo-EM reconstruction aligned with the Trs31 subunit from pdb:3CUE.
F Trs33 subunit from our cryo-EM reconstruction aligned with the TRAPPC6 (Trs33) subunit from pdb:2C0J.
G Trs20 subunit from our cryo-EM reconstruction aligned with the Sedlin (TRAPPC2, Trs20) subunit from pdb:1H3Q.
H Rab1/Ypt1 subunit from our cryo-EM reconstruction aligned with the Rab1/Ypt1 subunit from pdb:3CUE.

© 2021 The Authors

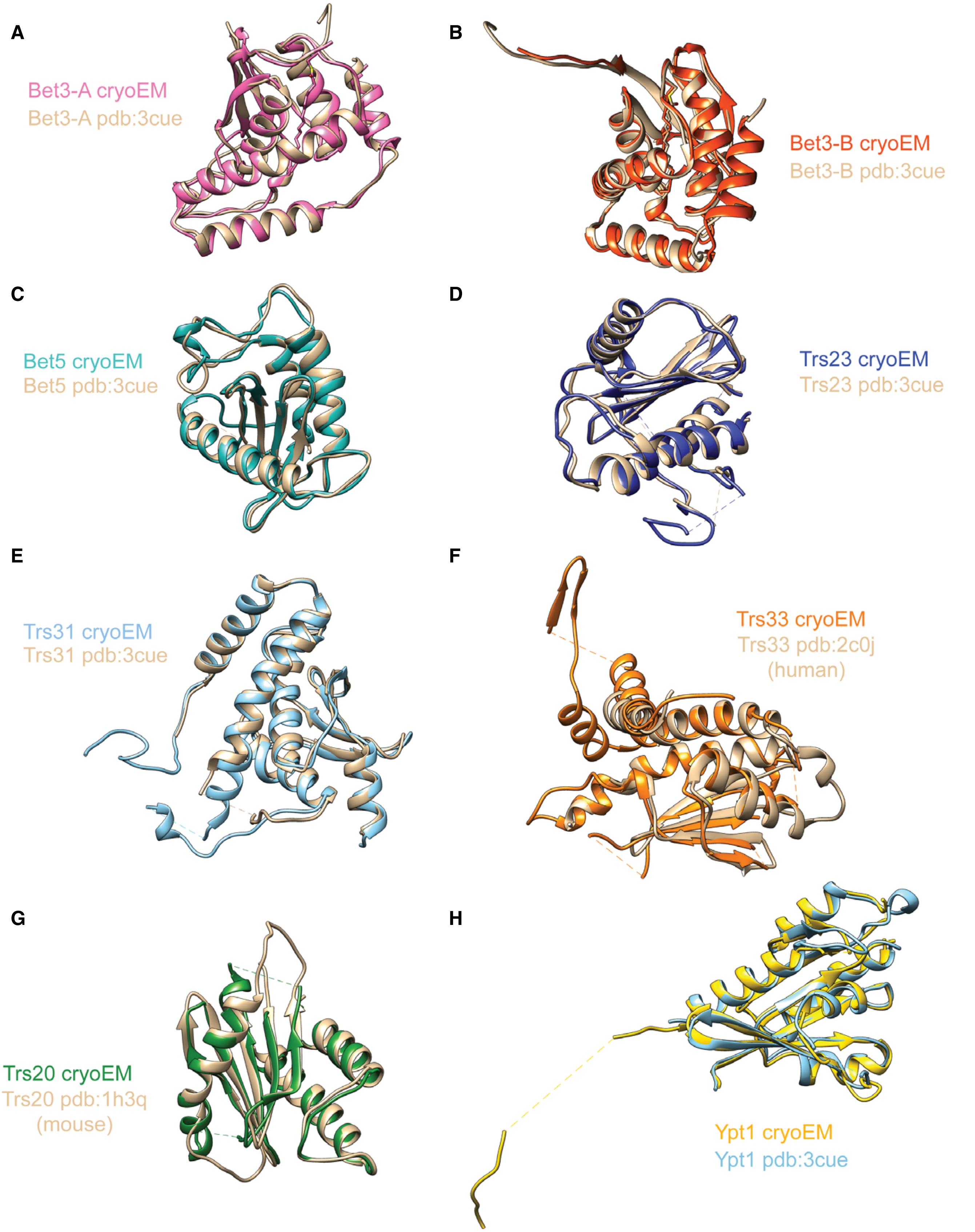

Figure EV4.

© 2021 The Authors

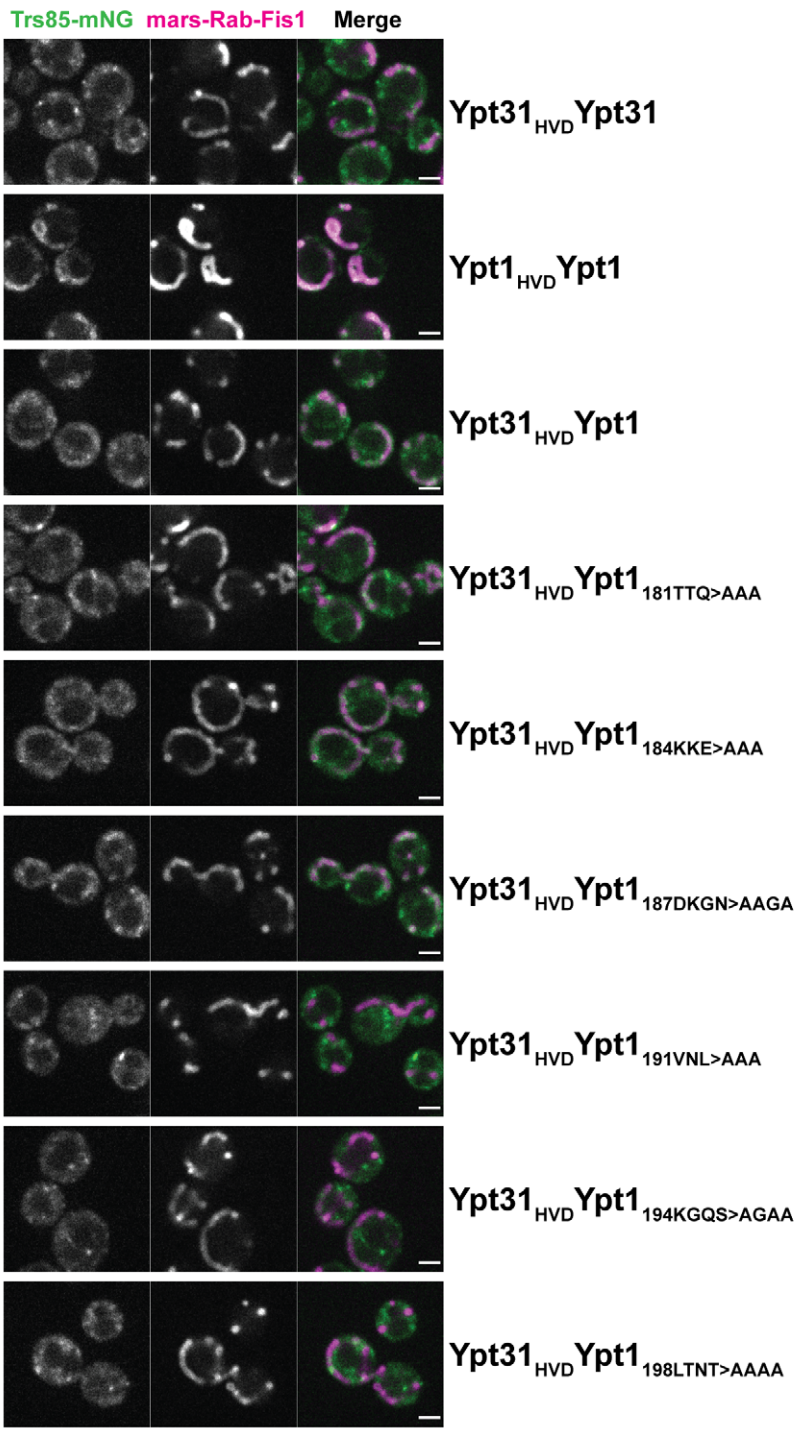

Figure EV5. Anchor-away of TRAPPIII to the mitochondria by nucleotide-free Rabs.

Live cell fluorescence microscopy of Trs85-mNeonGreen colocalization with mitochondrial-anchored mRFPmars-Rab substrates. Scale bar = 2 μm. Representative of n = 3 independent experiments.

© 2021 The Authors
