# Supplementary figures and images for: Structural basis of TRAPPIII‐mediated Rab1 activation
Source: EMBO J. 2021 May 21;40(12):e107607. doi: 10.15252/embj.2020107607 (PMC8204860; doi:10.15252/embj.2020107607)

From Figure panel 1D

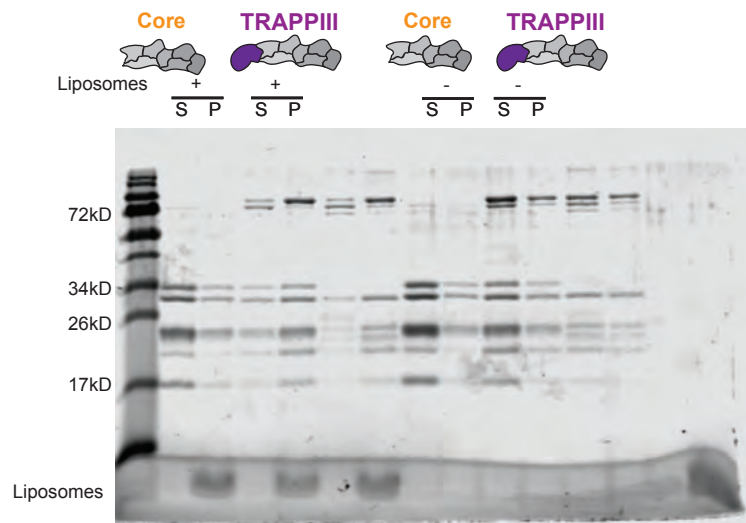

Supplement: Supplementary file 5 — Source Data for Figure 1 [file EMBJ-40-e107607-s006.pdf]

From Figure panel 6F

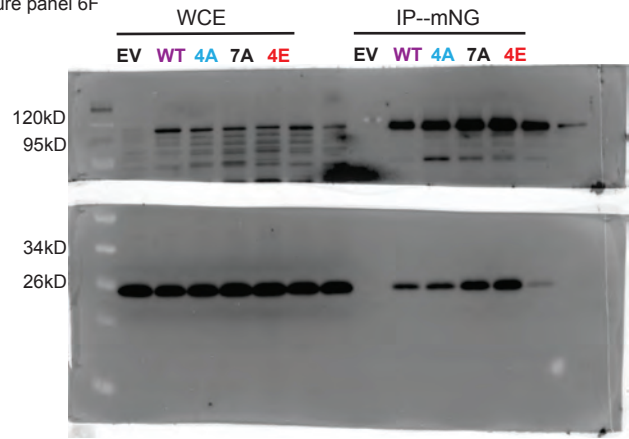

From Figure panel 6G

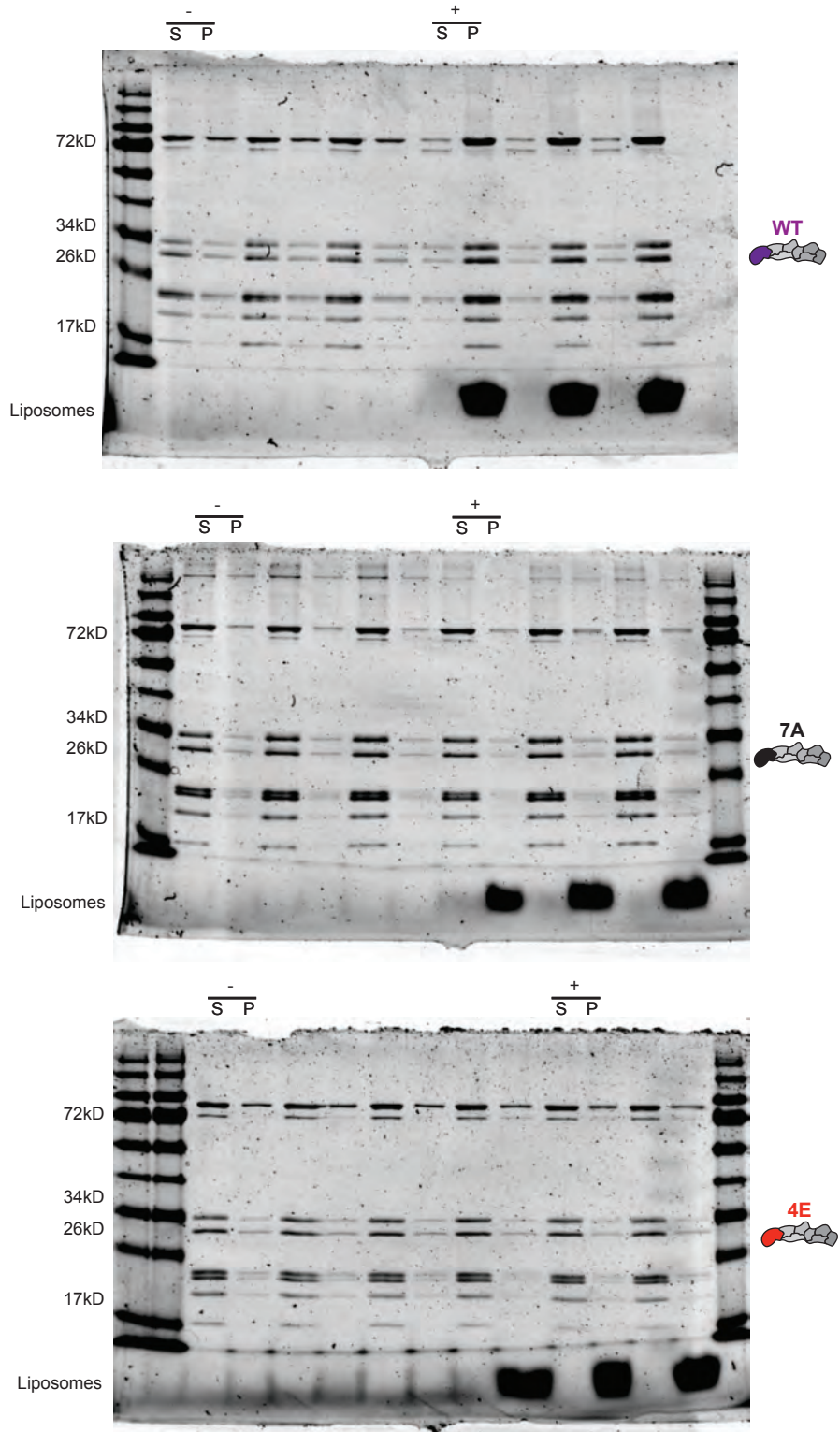

Supplement: Supplementary file 7 — Source Data for Figure 6 [file EMBJ-40-e107607-s005.pdf]

Core binding mutations

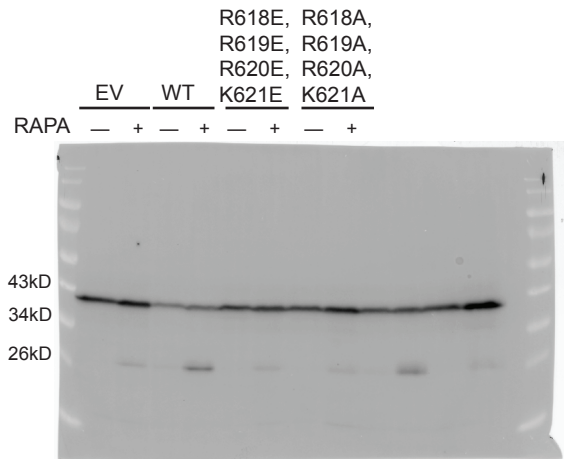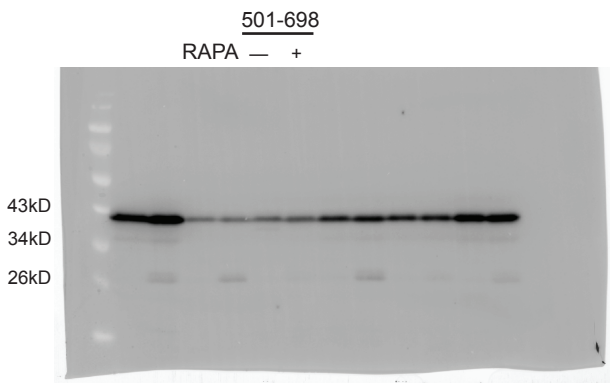

Membrane binding mutations

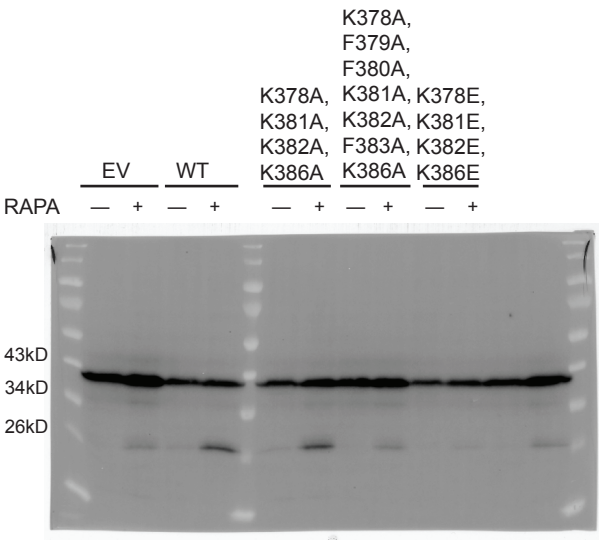

Supplement: Supplementary file 8 — Source Data for Figure 7 [file EMBJ-40-e107607-s007.pdf]
