# Supplementary material for: Structural basis of TRAPPIII‐mediated Rab1 activation
Source: EMBO J. 2021 May 21;40(12):e107607. doi: 10.15252/embj.2020107607 (PMC8204860; doi:10.15252/embj.2020107607)
Supplement: Supplementary file 6 — Source Data for Figure 4 [file EMBJ-40-e107607-s002.pdf]

From Figure panel 4A

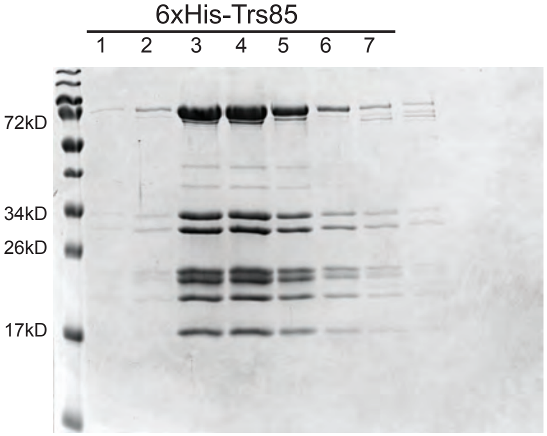

From Figure panel 4B

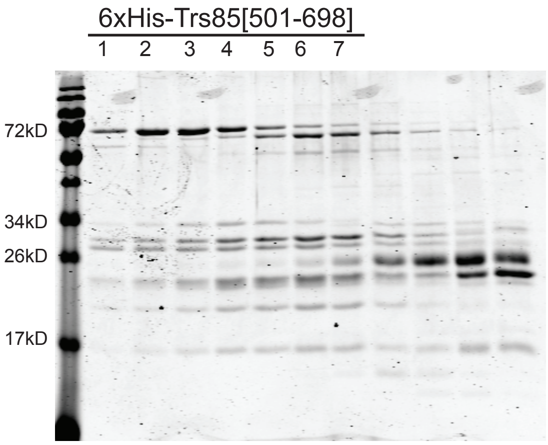

From Figure panel 4C

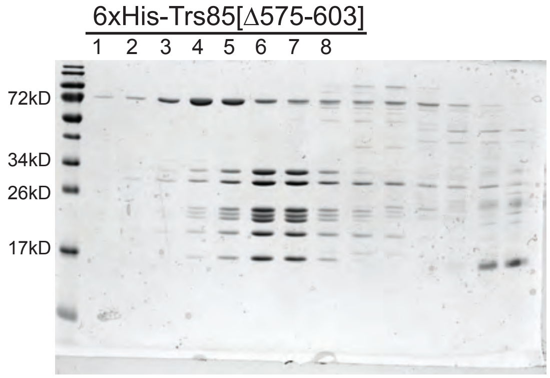

From Figure panel 4G

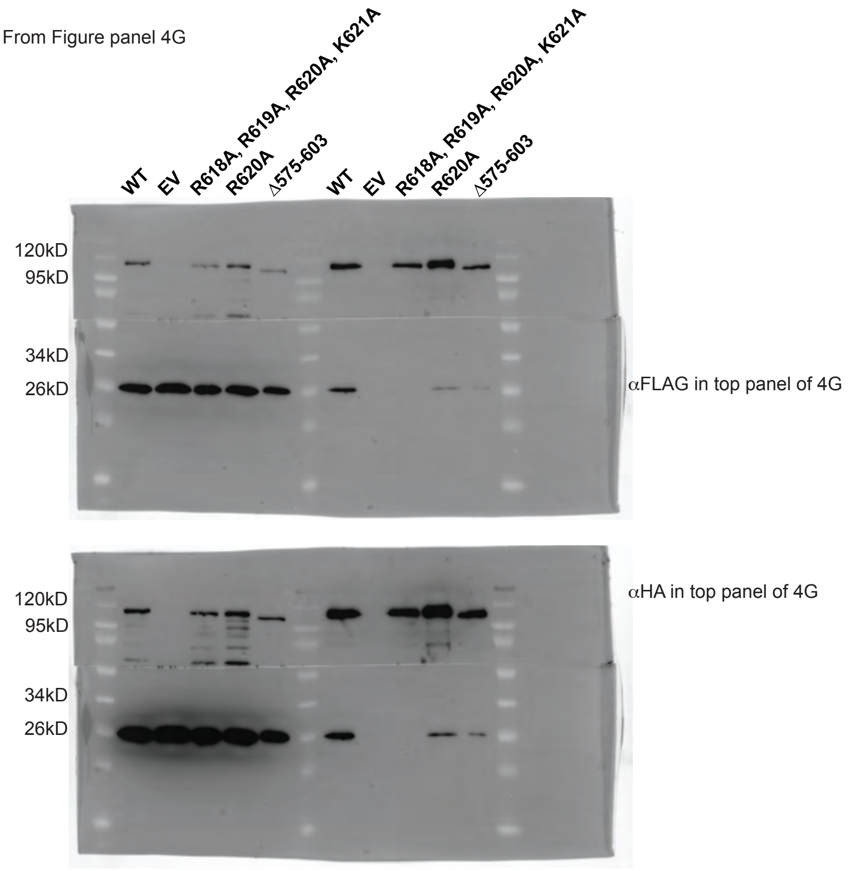

Same blot, multiple exposures.

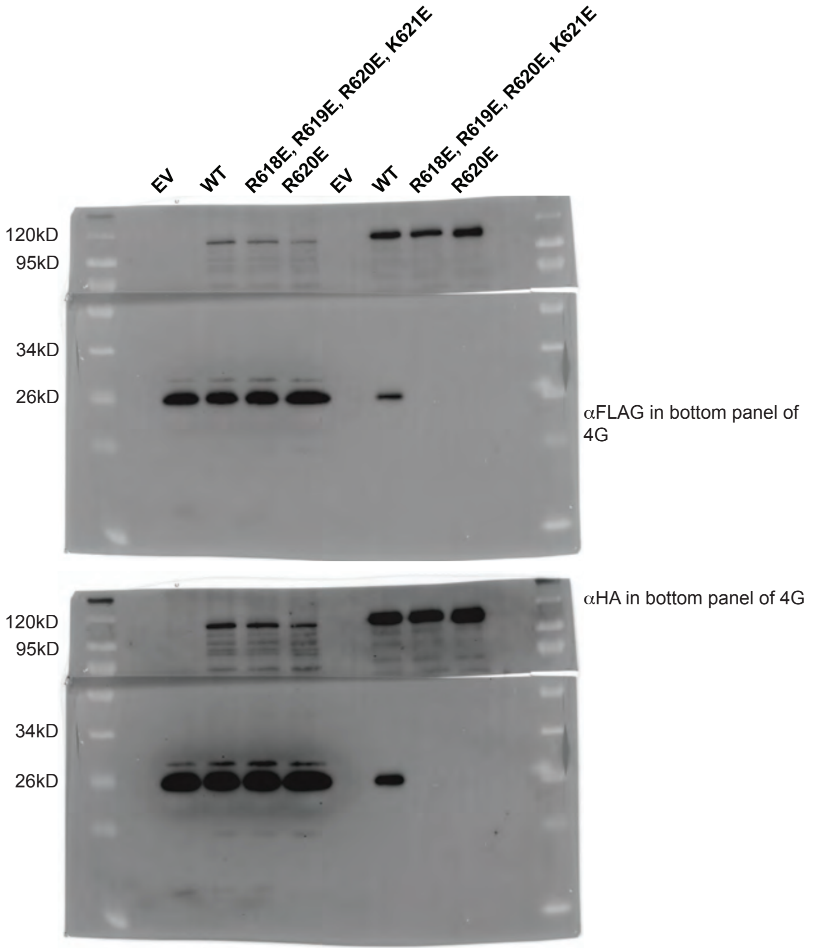

Same blot, multiple exposures.
